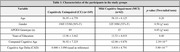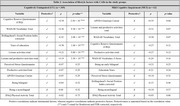# Identification of Lifestyle Factors Conditioning Cognitive Aging using Age Prediction Modeling

**DOI:** 10.1002/alz70856_105593

**Published:** 2026-01-09

**Authors:** Ainara Estanga, Iñigo Tellaetxe Elorriaga, Miren Altuna, Mirian Ecay‐Torres, Maite Garcia‐Sebastian, Asier Erramuzpe, Pablo Martinez‐Lage

**Affiliations:** ^1^ CITA Alzheimer Foundation, Donostia ‐ San Sebastián, Gipuzkoa, Spain; ^2^ University of the Basque Country, Leioa, Bizkaia, Spain; ^3^ Biobizkaia HRI, Barakaldo, Bizkaia, Spain; ^4^ Ikerbasque, the Basque Foundation for Science, Bilbao, Bizkaia, Spain

## Abstract

**Background:**

Cognitive aging describes changes in thinking, learning, and memory abilities as people age. While chronological age measures time lived, cognitive age reflects mental function, influenced by health, education, lifestyle, and social factors. The aims of this study were: to estimate individual's cognitive age based on neuropsychological test results; to determine the cognitive age delta (CAD, difference between an individual's predicted and actual chronological age) as a measure of cognitive aging; to examine the association between cognitive aging and lifestyle factors to identify potential contributors to cognitive maintenance with aging.

**Method:**

Cross‐sectional study. Population‐based recruitment from cohort with extensive clinical‐biological phenotyping (Gipuzkoa‐Alzheimer‐Project ‐PGA‐) in the Basque Country. CADs were computed using a Multiple Linear Regression model on neuropsychological test results. *Deltas* were compared between cognitively unimpaired (CU) and mildly cognitively impaired (MCI) participants using t‐tests, and analyzed correlations with factors including APOE4 genotype, bilingualism, years of education, vocabulary WAIS‐III as an estimate of intellectual level, cognitive reserve questionnaire (CRQ), and leisure and productive activity participation, perceived stress, and physical activity. Analyses used AgeML, an open‐source Python package.

**Result:**

Sample of 411 participants: 62 MCI individuals (average age 58±8 years, 52% female) and 349 CU individuals (average age 57±7 years, 56% female; see Table‐1 for participant characteristics). MCI subjects showed higher Cognitive Age (mean difference +3.82 years). Correlated factors with aging in CU and MCI participants are in Table‐2. In the CU group, CRQ score, vocabulary WAIS‐III score participation in leisure and productive activities, years of education and the Hollingshead Social position index were significantly correlated with CAD. In the MCI group, the differences for lifestyle factors were not statistically significant after Bonferroni and FDR correction, though APOE4 carrier status had a detrimental effect in the sample.

**Conclusion:**

We found factors of interest related to intellectual and social activities to target in the design of lifestyle interventions to maintain cognitive health and slow cognitive aging, with a particular focus on CU individuals. These findings are consistent with previous studies and reinforce the need to target the identified lifestyle factors.